# The first complete chloroplast genome sequence and phylogenetic analysis of *Actinidia trichogyna*

**DOI:** 10.1080/23802359.2026.2635831

**Published:** 2026-02-27

**Authors:** Sen Meng, Zheng Jiang, Jingwen Huang, Xiaohong Yao

**Affiliations:** a TaiShun Forestry Development Center, Taishun, China; b China Three Gorges University, Yichang, China; c Wuhan Botanical Garden, Chinese Academy of Sciences, Wuhan, China; d University of Chinese Academy of Sciences, Beijing, China

**Keywords:** *Actinidia trichogyna*, chloroplast genome, phylogenetic analysis

## Abstract

*Actinidia trichogyna* is primarily distributed in the high-altitude regions of southwest China and its genomic data resources are still lacking. In this study, we assembled and annotated its complete chloroplast genome, which spanned 156,507 bp and consisted of a large single-copy region of 88,295 bp, a small single-copy region of 20,610 bp and two inverted repeat regions of 23,801 bp. The genome contained 131 genes, including 84 protein-coding genes, 8 ribosomal RNA genes, and 39 transfer RNA genes. Phylogenetic analysis revealed that it formed an independent branch to other species in the Sect. *Maculatae*, suggesting its unique evolutionary history. This study provides valuable data resources for kiwifruit evolutionary and functional genomic studies.

## 1. Introduction

The genus *Actinidia* is one of the most important fruit trees, gathering significant attention due to its rich nutritional value, particularly its high vitamin C content (Liang et al. 2020). According to the latest taxonomic revision, *Actinidia* currently comprises 75 species or variants, nearly all of which are endemic to China, except for *A. strigose* and *A. hypoleuca* (Huang et al. 2013). The abundant *Actinidia* germplasm resources in China lay a solid foundation for future insights into the speciation, evolution, and subsequent breeding of kiwifruit.

**Figure 1.**
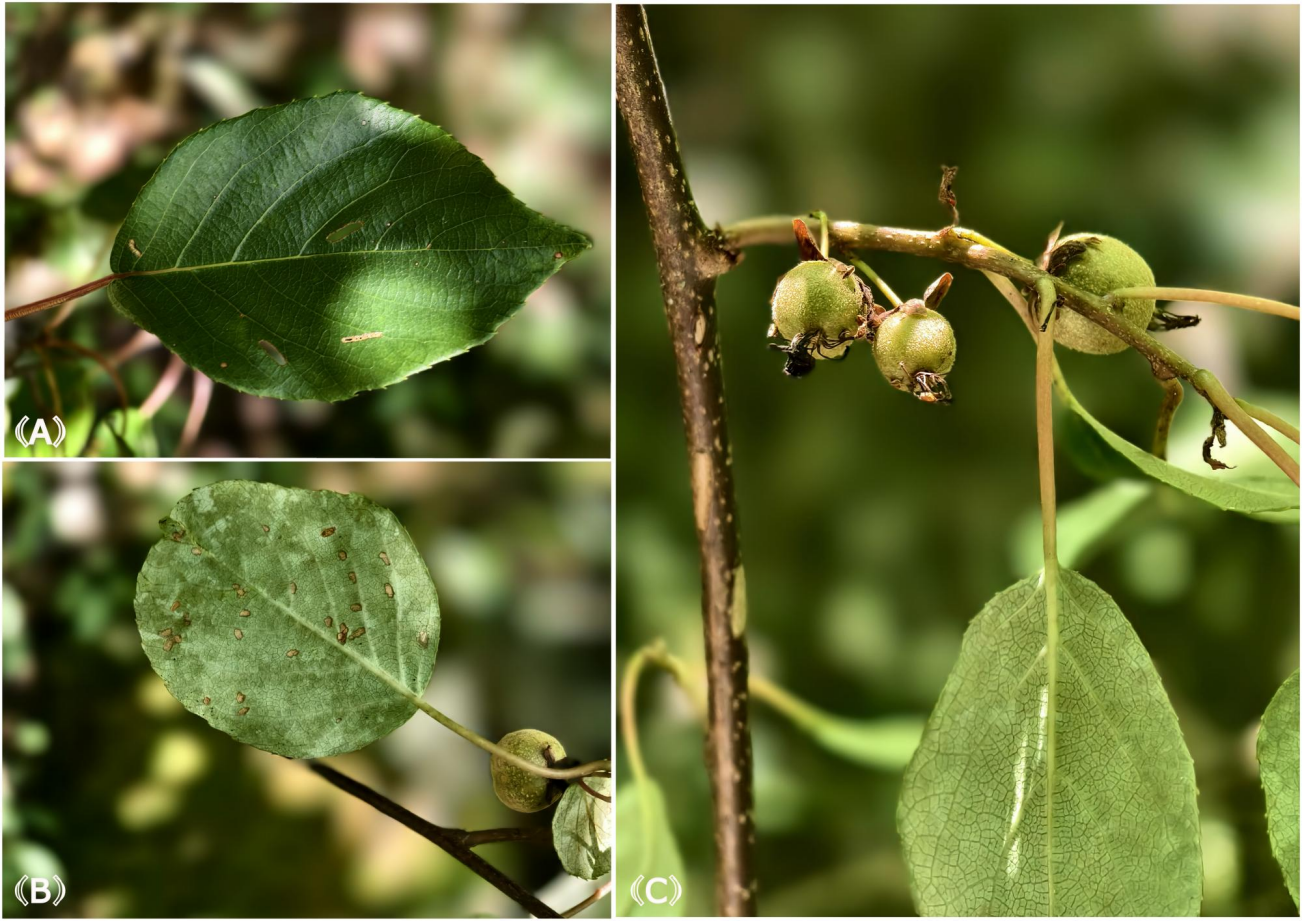
Morphological characteristics of *A. trichogyna*. Photographs were taken by Jingwen Huang in Wuxi County, Chongqing municipality, on August 1, 2024. (A) The adaxial leaf surface. Green and glabrous, midvein and lateral veins subconspicuous abaxially, lateral veins 6 or 7 pairs. (B) The abaxial leaf surface. Glaucous and glabrous, base usually truncate, margin finely serrulate, apex acute to acuminate. (C) The fruits and stem. Fruits subglobose, with lenticels, glabrescent at maturity. Stems dark brown, glabrous, with conspicuous lenticels.

*Actinidia trichogyna* Franch. (1894) is primarily distributed in the high-altitude regions of southwest China, such as Chongqing municipality, Sichuan province, and Hubei province (Li et al. 2007). The reference images of *A. trichogyna* are presented in Figure 1. According to the latest taxonomic revision, it is classified within the Sect. *Maculatae* and shares similar morphological characteristics with *A. chrysantha*, *A. glaucocallosa* and *A. indochinensis*. However, the phylogenetic relationships of *A. trichogyna* are still uncertain owing to lacking of genomic data resources, so it is crucial to fill the gap within *Actinidia*. The chloroplast genome is a key component of the plant plastid genetic system and contains relatively independent genetic information (Daniell et al. 2016). Due to its small genome size, relative conservation and low nucleotide substitution rates, it has been widely used in studying molecular evolution and phylogeographical history (Jansen et al. 2007). Moreover, a complete chloroplast genome serves as a crucial resource for studying the phylogenetic relationships and taxonomy within the genus *Actinidia*. Therefore, it is essential to explore the complete chloroplast genome sequence of *A. trichogyna*.

In this study, we sequenced, assembled, and annotated the complete chloroplast genome of *A. trichogyna*. Comparative analyses were conducted with other published chloroplast genomes in the NCBI database to explore the phylogenetic relationships of *A. trichogyna*. Our study provides valuable genomic information for the species classification, conservation, and molecular breeding of kiwifruit.

## 2. Materials and methods

### 2.1. Plant sampling, DNA extraction and sequencing

The leaves of *A. trichogyna* for this study were collected from the Wuxi County, Chongqing municipality (109.88°E, 31.47°N, altitude. 1592 m), in August 2024. The fresh leaves were collected and preserved in silica gel. The voucher specimen (voucher: HIB0258940; contact person: Guangwan Hu, guangwanhu@wbgcas.cn) was identified and deposited at the Herbarium of Wuhan Botanical Garden, Chinese Academy of Sciences on September 12, 2025.

Paired-end sequencing libraries were constructed following the manufacturer’s protocol on the DNBSEQ-T7 platform by Novogene (Beijing, China), with insert sizes of approximately 150 bp. Low-quality reads and adapter sequences were removed from the raw sequencing data using fastp v0.23.2 (Chen et al. 2018).

### 2.2. Assembly and annotation of the chloroplast genome

Approximately 7 GB of clean reads of *A. trichogyna* were produced and further evaluated using FastQC v0.11.9 (Brown et al. 2017). The de novo plastome assembly was performed by GetOrganelle v1.7.5 with parameter ‘-R 15 -t 20 -k 21,45,65,85,105 -F embplant_ pt’ (Jin et al. 2020). The genome sequence was annotated by using PGA 2.0 (Zhang et al. 2025) and CPGAVAS2(Shi et al. 2019) and then manually merged and modified, with *Actinidia chinensis* Planch. (GenBank Accession no. NC_026690) as the reference genome. The assembled chloroplast genome map was generated using OGDRAW v1.3.1 (Greiner et al. 2019).

### 2.3. Phylogenetic analysis

We downloaded 26 cp genome sequences of other *Actinidia* species from the NCBI database, and *Clematoclethra scandens* subsp*. hemsleyi* (Actinidiaceae) was selected as the outgroup. All the cp genome sequences were aligned using MAFFT v.7 (Nakamura et al. 2018), and the poorly aligned regions were then removed using trimAl v1.2 (Capella-Gutierrez et al. 2009). The Maximum-likelihood (ML) tree was reconstructed in IQ-TREE v2.0.3 (Bui et al. 2020) with rapid 1000 bootstrap replicates. The phylogenic tree was then visualized by the online website Chiplot (Xie et al. 2023).

## 3. Results

### 3.1. Characteristics of chloroplast genome

**Figure 2.**
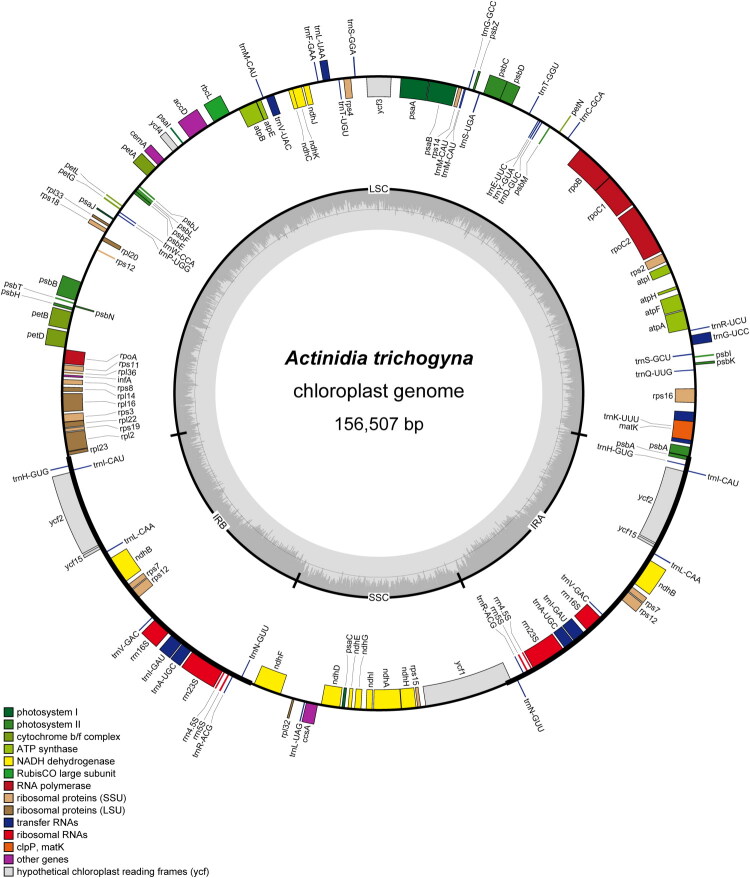
The circular chloroplast map of *A. trichogyna*. The distribution of genes are shown on the outermost track. The genes categories are shown in the bottom left corner. The inner circle represents a typical quadripartite structure with a large single-copy (LSC) region, a pair of inverted repeats (IRa and IRb) regions and a small single-copy (SSC) region.

The average coverage depth of the assembled genome was 11091.8× (Figure S1). The complete cp genome of *A. trichogyna* was 156,507 bp in length and showed a typical quadripartite structure, consisting of a large single-copy (LSC) region of 88,295 bp, a small single-copy (SSC) region of 20,610 bp and a pair of inverted repeat (IRs) regions of 23,801 bp (Figure 2). The genome had an overall GC content of 37.2%, with higher GC content observed in the IR regions (43.2%) compared to the LSC (35.4%) and SSC (31.1%) regions. The annotation identified a total of 131 genes, including 84 protein-coding genes (PCGs), 8 ribosomal RNA genes (rRNAs), and 39 transfer RNA genes (tRNAs). Among them, 12 genes (*rps*16, *atp*F, *rpo*C1, *ycf*3, *pet*B, *pet*D, *rpl*16, *rpl*2, *ndh*B, *ndh*A, *ycf*1, and *ndh*B) were cis-splicing genes (Figure S2), and *rps*12 was a trans-splicing gene with three unique exons (Figure S3). The newly completed annotation of the *A. trichogyna* chloroplast genome has been deposited to GenBank under the accession number PX754132.

### 3.2. Phylogenetic relationship

**Figure 3.**
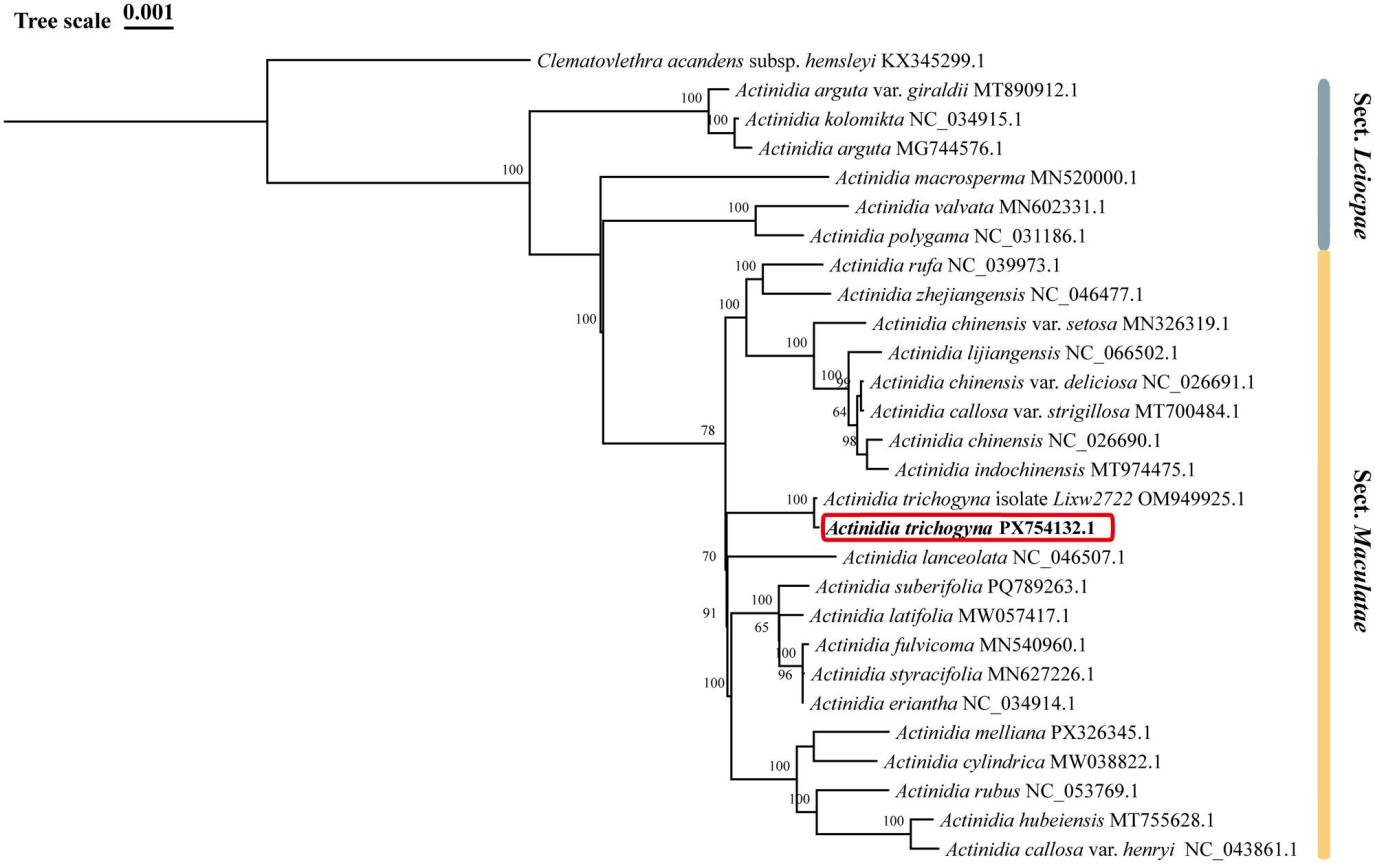
The maximum-likelihood tree based on the chloroplast gene sequences of *A. trichogyna* and other 25 species of *Actinidia*. The number next to the nodes indicates the bootstrap values. The scale bar in the top left corner of the figure represents the phylogenetic distance of 0.001 nucleotide substitutions per site. The following sequences are used: *A. arguta* MG744576.1 (Lin et al. 2018), *A. arguta* var. *giraldii* MT890912.1 (Ding et al. 2021), *A. callosa* var. *henryi* NC_043861.1 (Wu et al. 2019), *A. callosa* var. *strigillosa* MT700484.1 (Liu et al. 2020), *A. chinensis* NC_026690.1 (Yao et al. 2015), *A. chinensis* var. *deliciosa* NC_026691.1 (Yao et al. 2015), *A. chinensis* var. *setosa* MN326319.1 (Lin et al. 2019), *A. cylindrica* MW038822.1 (Ma and Liu 2019), *A. eriantha* NC_034914.1 (Tang et al. 2019), *A. fulvicoma* MN540960.1 (Zhang et al. 2019), *A. hubeiensis* MT755628.1, *A. indochinensis* MT974475.1, *A. kolomikta* NC_034915.1 (Lan et al. 2018), *A. lanceolata* NC_046507.1 (Zhang and Liu 2019), *A. latifolia* MW057417.1 (Yang et al. 2021), *A.lijiangensis* NC_066502.1 (Lin et al. 2025), *A. macrosperma* MN520000.1 (Chen et al. 2019), *A. melliana* PX326345.1, *A. polygama* NC_031186.1 (Wang et al. 2016), *A. rubus* NC_053769.1 (Xu et al. 2020), *A. rufa* NC_039973.1 (Kim et al. 2018), *A. styracifolia* MN627226.1 (Yang et al. 2019), *A. suberifolia* PQ789263 (Zhang et al. 2025), *A. trichogyna* PX754132, *A. trichogyna* isolate Lixw2722 OM949925.1, *A. valvata* MN602331.1 (Chen et al. 2020), *A. zhejiangensis* NC_046477,1 (Ai and Liu 2019), *Clematoclethra scandens* subsp. *hemsleyi* KX345299.1 (Wang et al. 2016).

Phylogenetic reconstruction using Maximum Likelihood (ML) methods robustly revealed the position of *A. trichogyna* within the genus based on 28 whole chloroplast genome sequences of *Actinidia* and the outgroup (Figure 3). In the phylogenetic tree, 26 *Actinidia* species clustered into two main clades with high bootstrap support, closely consistent with the latest taxonomic revision. In the first main clade, one branch contained *A. arguta*, *A. arguta* var. *giraldii* and *A. kolomikta*, and another one contained *A. macrosperma*, *A. polygama*, *A. valvata*, which corresponded to the Ser. *Lamellatae* and Ser. *Solidae* of Sect. *Leiocarpae*, respectively. The rest species clustered into another main clade, which were in correspondence with the Sect. *Maculatae*. Phylogenetic analysis indicated that *A. trichogyna* did not cluster with other species, suggesting its distinct evolutionary history. Besides that, *A. chinensis*, *A. chinensis* var. *deliciosa*, *A. lijiangensis*, *A. chinensis* var. *setosa, A. indochinensis* and *A. callosa* var. *strigillosa* clustered together, whereas *A. zhejiangensis* and *A. rufa* formed another independent cluster*. A. suberifolia*, *A. latifolia*, *A. eriantha*, *A. styracifolia* and *A. fulvicoma* clurested together, while *A. melliana, A. cylindrica*, *A. rubus*, *A. callosa* var. *henryi* and *A. hubeiensis* showed closely phylogenetic relationships in another branch.

## 4. Discussion and conclusion

This study reported the complete chloroplast genome of *A. trichogyna* and analyzed its structural features and gene content, which was similar to other previously reported species of *Actinidia*. The genome showed a typical quadripartite structure spanning 156,507 bp, which consisted of two inverted repeats (IRs) of 23,801 bp separated by a large single-copy (LSC) and a SSC of 88,295 bp and 20,610 bp. The phylogenetic analysis based on 25 whole chloroplast genome sequences revealed the phylogenetic position of *A. trichogyna* within the genus, which displayed consistent topology with the previous reports (Liu et al. 2017; Wang et al. 2022). The two chloroplast genome sequences of *A. trichogyna* (Accessions: PX754132.1 and OM949925,1) clustered together with a 100% bootstrap value in the phylogenetic analysis, which confirmed the accuracy of our assembly and the sequence conservation of the species’ plastome. The phylogenetic results indicated that *A. trichogyna* formed an independent lineage parallel to other species in the Sect. *Maculatae*. Based on morphological characteristics, *A. trichogyna* was close to *A. indochinensis* but not supported by the phylogenetic analysis (Figure 3). Therefore, further studies are needed to resolve the phylogenetic relationships within the genus *Actinidia*.

The complete chloroplast genome and phylogenetic analysis of *A. trichogyna* provided valuable perspectives for clarifying the evolutionary relationships within the genus *Actinidia*. Future investigations should combine more extensive species sampling and nuclear data, which will enhance our comprehension of molecular evolution and species conservation for this economically important genus.

## Supplementary Material

supplementary material.docx

## Data Availability

The complete chloroplast genome sequence of *A. trichogyna* in this study has been submitted to the NCBI database under the accession number PX754132. The associated BioProject, BioSample and SRA numbers are PRJNA1398415, SAMN54450834 and SRX31691751, respectively.
