# Supplementary material for: The first complete chloroplast genome sequence and phylogenetic analysis of Actinidia trichogyna
Source: Mitochondrial DNA B Resour. 2026 Feb 27;11(4):462–7. doi: 10.1080/23802359.2026.2635831 (PMC12951675; doi:10.1080/23802359.2026.2635831)
Supplement: supplementary material.docx [file TMDN_A_2635831_SM6719.docx]

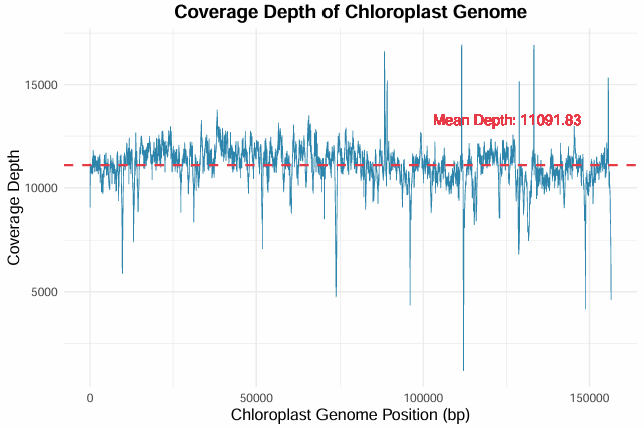


**Figure S1.** The sequencing depth and coverage map of the chloroplast genome of *Actinidia trichogyna*. The map illustrates the sequencing depth distribution across the chloroplast genome, with a total length of 156,507bp and an average sequencing depth of 11091.8×. The X and Y axes represent the nucleotide position and the corresponding coverage depth, respectively.


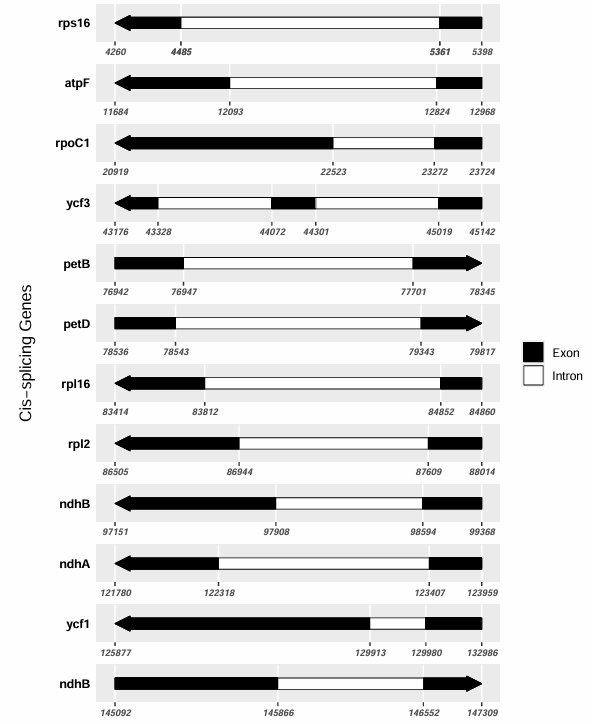


**Figure S2.** The schematic map of the cis-splicing genes in the chloroplast genome of *Actinidia trichogyna*. Gene names appear on the left side, and the corresponding structures are displayed on the right. Exons and introns are respectively shown in black and white. The arrow indicates the sense direction of the gene.


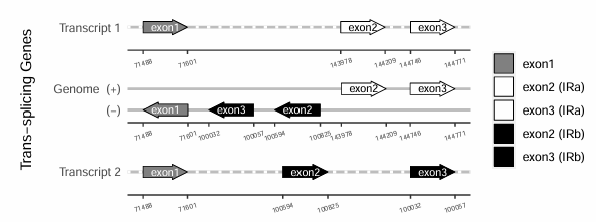


**Figure S3.** The schematic map of the trans-splicing gene *rps*12 in the chloroplast genome of *Actinidia trichogyna*. It has three unique exons. Two of them are duplicated as they are located in the IR regions.
